# Understanding anti-vascular endothelial growth factor medications: chemical properties, costs, and side effects

**DOI:** 10.25122/jml-2025-0148

**Published:** 2026-07

**Authors:** Hanan Makhdoum, Abeer Alharbi, Shaden Alhazmi, Ahlam Alrehaili, Rahaf Afandi, Ghadi Aljohani, Khalid Alharbi

**Affiliations:** 1 Department of Ophthalmology, College of Medicine, Taibah University; 2 College of Medicine, Taibah University, Medina, Saudi Arabia; 3 Department of Ophthalmology, Ohud Hospital, Madinah, Saudi Arabia

**Keywords:** anti-VEGF, retinal diseases, faricimab, ranibizumab, bevacizumab, AMD, age-related macular degeneration, Ang-2, angiopoietin-2, CAD, Canadian dollar, DME, diabetic macular edema, DR, diabetic retinopathy, ETDRS, Early Treatment Diabetic Retinopathy Study, Fab, fragment antigen-binding, FDA, Food and Drug Administration, GFR, glomerular filtration rate, ICER, incremental cost-effectiveness ratio, IOP, intraocular pressure, nAMD, neovascular age-related macular degeneration, PlGF, placental growth factor, QALY, quality-adjusted life year, RRD, rhegmatogenous retinal detachment, scFv, single-chain variable fragment, VEGF, vascular endothelial growth factor

## Abstract

Vascular endothelial growth factor (VEGF) is an important driver of abnormal retinal neovascularization and increased vascular permeability. VEGF overexpression is associated with a number of retinal conditions, such as diabetic macular edema (DME), and neovascular age-related macular degeneration (nAMD). Anti-VEGF treatments have been the most common for these conditions and have improved vision by reducing abnormal blood vessel formation and leakage. This narrative review addressed the fact that the pharmacological properties, safety profiles, and cost-effectiveness of the currently marketed anti-VEGF intravitreal agents (i.e. bevacizumab, ranibizumab, aflibercept, brolucizumab, pegaptanib, and recently faricimab) differ and therefore, each of these agents, have their own unique molecular structure, target specificity, duration of action in the eye, and systemic circulation, each of which will affect the dosing intervals and treatment burden as well as the safety and adverse events associated with each drug. Aflibercept is a high-binding-affinity decoy receptor and is therefore distinct from faricimab, which is a dual-action (VEGF-A and angiopoietin-2) inhibitor. By contrast, ranibizumab, bevacizumab, brolucizumab, and pegaptanib are anti-angiogenic agents with different VEGF blockade profiles, which ultimately lead to distinct clinical outcomes and pharmacokinetic profiles. While anti-VEGF therapies are highly effective, the outcomes regarding safety, efficacy, and cost depend heavily on the study design and reflect the actual treatment paradigm and continuum of care within the healthcare system. The various attributes of agents should help personalize a patient’s treatment approach while also addressing potential challenges related to the safety, efficacy, and longevity of treatment for retinal diseases.

## INTRODUCTION

Vascular endothelial growth factor (VEGF) is a highly specific endogenous growth factor that promotes the development of vascular endothelial cells. Under normal physiological conditions, VEGF supports endothelial cell regeneration, facilitating the formation of functional blood vessels without causing vascular leakage [1]. However, in certain pathological states, such as inflammatory, neoplastic, or ischemic conditions, this process becomes dysregulated. VEGF can then drive abnormal neovascularization, forming new, often fragile, and leaky blood vessels, and promoting breakdown of the blood-retinal barrier, with subsequent intraretinal and subretinal fluid accumulation that contributes directly to visual loss [2]. Several retinal diseases driven by excessive neovascularization, including age-related macular degeneration (AMD), diabetic retinopathy, and retinal vein occlusion, are significant causes of vision loss worldwide. These conditions underscore the critical role of VEGF in the pathological mechanisms that threaten visual health [3].

Anti-VEGF therapies target and inhibit VEGF activity, preventing the growth of abnormal blood vessels and reducing vascular leakage in the retina. This intervention plays a key role in preserving central vision and improving visual outcomes for patients with these serious retinal disorders [4]. Nonetheless, the currently available anti-VEGF agents differ in molecular structure, target specificity, intravitreal durability, systemic exposure, and dosing interval, which could affect efficacy, treatment burden, safety, and cost in real-world clinical practice [5,6]. With the expansion of the therapeutic arsenal from selective VEGF inhibition to wider ligand blockade and dual-pathway inhibition, the need for comparative assessment has become more pertinent. As such, this narrative review aimed to provide a comprehensive overview of the chemical structures and pharmacokinetics of various anti-VEGF drugs and assess the pharmacologic characteristics, relative safety, and cost analyses of the primary anti-VEGF agents used in retinal disease, focusing on clinically significant differences in mechanisms, durability, treatment burden, and ease of use.

## Material and Methods

### Literature search strategy

A literature search for relevant studies regarding the use of anti-VEGF therapy for retinal diseases was conducted across PubMed, Scopus, Web of Science, and Cochrane Library from their inception until January 2025. The search strategy consisted of free-text keywords and indexed terms that were relevant to the biology of VEGF, retinal vascular disorders, and specific anti-VEGF agents. The primary keywords were ‘VEGF’, ‘vascular endothelial growth factor’, ‘faricimab’, ‘aflibercept’, ‘ranibizumab’, ‘bevacizumab’, ‘brolucizumab’, ‘pegaptanib’, ‘neovascular age-related macular degeneration’, ‘diabetic macular edema’, ‘diabetic retinopathy’, ‘retinal vein occlusion’, ‘pharmacokinetics’, ‘pharmacodynamics’, ‘safety’, ‘adverse events’, ‘cost-effectiveness’, ‘cost utility’, and ‘economic evaluation’. The reference lists of the major reviews and the key articles were screened for additional studies.

### Eligibility criteria

Eligible sources included peer-reviewed randomized controlled trials, comparative clinical studies, systematic reviews, meta-analyses, and large observational studies, as well as pharmacokinetic and preclinical studies on ophthalmic drug behavior, instructions for use, and economic studies on anti-VEGF treatment for retinal diseases. Publications in languages other than English were excluded. Editorials, narrative commentaries, duplicate publications, and conference abstracts, posters, and preprints were excluded when a peer-reviewed full-text version was available.

### Study selection and source prioritization

The chosen studies were assessed concerning the objectives of the review and classified based on the following criteria: (1) molecular composition and mechanisms of action, (2) pharmacology and durability of therapeutic response, (3) safety, both ocular and systemic, (4) financial aspects, including the burden of treatment and cost-effectiveness. In cases of multiple publications on the same subject, priority was given to randomized studies, systematic reviews, meta-analyses, and the most robust comparative studies.

### Economic evidence

Economic results were interpreted with caution, as direct comparisons of studies may be affected by variations in healthcare systems, currencies, reimbursement systems, analytical perspectives, and assumptions about injection frequency. Therefore, economic evidence was used to demonstrate context-specific considerations rather than a claim of cost-effectiveness rankings across agents.

## Results

### Comparative molecular design and pharmacodynamic implications of anti-VEGF agents

The current intravitreal anti-VEGF agents are best compared in terms of molecular scaffold, target spectrum, and pharmacodynamic effects, rather than single-agent descriptions. While faricimab, aflibercept, ranibizumab, bevacizumab, brolucizumab, and pegaptanib all inhibit pathological VEGF activity, they vary widely in molecular structure, ligand binding, systemic distribution, and the durability of treatment effects [7–11]. These characteristics are important in clinical practice, particularly regarding treatment interval, treatment burden, anatomical response, and safety in routine retinal practice (Table 1).

**Table 1:** Comparative molecular and pharmacodynamic features of current anti-VEGF agents

Agent	Molecular scaffold/approximate MW	Target spectrum	Standard intravitreal dose used clinically	Durability signal from pivotal studies	Main pharmacodynamic implication
**Faricimab**	Bispecific humanized IgG1 CrossMAb, ~149 kDa; Fc region engineered to abolish Fcγ/FcRn binding	VEGF-A + Ang-2	6 mg / 0.05 mL	Phase 2 STAIRWAY supported q12–q16w dosing vs monthly ranibizumab; phase 3 TENAYA/LUCERNE and YOSEMITE/RHINE confirmed dosing up to q16w in selected patients	Dual-pathway inhibition aims to improve vascular stability and reduce treatment burden
**Brolucizumab**	Humanized single-chain Fv (scFv), ~26 kDa	VEGF-A	6 mg / 0.05 mL	HAWK/HARRIER showed noninferior vision vs aflibercept, with >50% of 6-mg eyes maintained on q12w through week 48; label allows q8–12w after loading	Small size permits high molar dose per injection and may support deeper tissue penetration/durability
**Pegaptanib**	Pegylated RNA aptamer, ~50 kDa	Selective VEGF165 isoform	0.3 mg every 6 weeks	VISION/NEJM trial established benefit over sham on fixed q6w dosing	Historically important proof-of-concept, but a narrower blockade than later agents
**Aflibercept**	Recombinant fusion decoy receptor, total ~115 kDa (97-kDa protein core plus glycosylation)	VEGF-A, VEGF-B, PlGF	2 mg / 0.05 mL	VIEW studies supported q8w dosing after loading, with similar visual outcomes to monthly ranibizumab	Broader ligand trapping and high binding affinity support durable VEGF suppression
**Ranibizumab**	Humanized Fab fragment, ~48 kDa, Fc-free	VEGF-A	0.5 mg / 0.05 mL for adult nAMD/RVO; 0.3 mg / 0.05 mL for DME in US labeling	MARINA/ANCHOR established strong efficacy with monthly dosing; label notes reduced efficacy with much less frequent dosing	Fc-free design favors ocular diffusion and relatively low systemic exposure
**Bevacizumab**	Full-length humanized IgG1 antibody, ~149 kDa	VEGF-A	Common ophthalmic dose 1.25 mg / 0.05 mL (off-label)	CATT/IVAN showed visual outcomes comparable to ranibizumab when matched by regimen in nAMD	Lowest-cost platform; full IgG structure contributes to greater systemic persistence than ranibizumab

**Abbreviations:** Ang-2, angiopoietin-2; FcRn, neonatal Fc receptor; MW, molecular weight; PlGF, placental growth factor; q8w/q12w/q16w, every 8/12/16 weeks. **Note:** Bevacizumab is listed with the commonly used intravitreal ophthalmic dose in comparative retinal trials and routine practice; this remains an off-label ocular use of the oncology antibody.

Molecularly speaking, faricimab is the most different from the other agents because it is the only bispecific antibody made via CrossMAb technology [12] and is meant to inhibit both VEGF-A and Angiopoietin-2 [12,13] and therefore integrates both of the opposing processes pertaining to vascular instability and the active pathology of disease in the eye [14]. By comparison, aflibercept is a recombinant fusion protein that acts as a high-affinity soluble decoy receptor [15–17] and therefore possesses greater functionality because it is capable of interacting with not only VEGF-A but also VEGF-B and placental growth factor, making it a more versatile agent than those that only act on the VEGF-A [15,17]. While ranibizumab is a humanized Fab fragment and is designed to be used intraocularly [9,18–20], and, on the other hand, bevacizumab is a full-length IgG antibody which was designed to be used in oncology and then later became widely used in ophthalmology because it was more cost-effective [10,21–23]. Moreover, brolucizumab is a single-chain antibody fragment, whereas pegaptanib is a pegylated aptamer selected for the VEGF165 isoform [11,24].

The structural changes mentioned affect the pharmacodynamics of the drugs. For instance, Ranibizumab’s smaller structure lacks an Fc domain and is designed to improve diffusion within the retina while limiting systemic exposure [9,25]. Bevacizumab, because it has a complete IgG structure, offers the benefit of VEGF-A inhibition but, unlike ranibizumab, exhibits greater systemic persistence following intravitreal injection [25]. With aflibercept, the combination of intermediate size, higher binding affinity, and broadened ligand capture helps to explain the persistence of intravitreal activity across varying treatment settings [8,15]. Faricimab takes this notion a step further by targeting VEGF-A and blocking angiopoietin-2, with phase II data indicating that this combination may provide sufficient disease control with less frequent injections, possibly even less than the monthly dosing of ranibizumab, in a limited population [12,26].

Brolucizumab exemplifies the balance between highly optimized molecular constructs and their clinical implications. Its single-chain antibody fragment enabled delivery of a high molar amount of anti-VEGF within a standard intravitreal injection volume, a feature associated with improved tissue penetration and possibly longer dosing intervals [27,28]. However, the discussion of durability must include safety, especially in light of recent literature on brolucizumab and retinal vasculitis and retinal vascular occlusion [29]. In contrast, pegaptanib is of virtually no value today, even though it was the first approved ocular VEGF antagonist and demonstrated clinical benefit compared with sham treatment. Its clinical value is diminished by selective inhibition of the VEGF165 isoform, and its pharmacologic scope is more limited than that of more modern agents with broader or dual-pathway blockade [11,24].

Currently available anti-VEGF agents should not be viewed as interchangeable, given their different pharmacology and chemistry. Instead, these agents represent different therapeutic mechanisms. These mechanisms include: selective aptamer inhibition with pegaptanib [11,24], VEGF- A blockade with ranibizumab [9], full-length antibody inhibition with bevacizumab [10], receptor-fusion ligand trapping with aflibercept [15], compact single-chain inhibition with brolucizumab [27,28], and dual-pathway bispecific inhibition with faricimab [12]. This sort of assessment provides clarity on how molecular characteristics may be responsible for differences in durability, systemic exposure, treatment burden, and clinically meaningful safety considerations (Table 1).

### Safety profiles

Ranibizumab, bevacizumab, aflibercept, faricimab, and brolucizumab are among the anti-VEGF medications currently used in retinal practice. Although randomized clinical trials have consistently shown that intravitreal injection therapy improves visual outcomes across several retinal diseases, treatments carry the risk of adverse events related to their mechanism of action and the injection itself [30]. The most common and initial indication for intravitreal anti-VEGF injections was neovascular age-related macular degeneration (nAMD), but there are now other approved indications, including diabetic macular edema and macular edema related to central and branch retinal vein occlusions. In addition, these medications are used off-label to mitigate the effects of numerous other retinovascular diseases, proliferative diabetic retinopathy, vitreous hemorrhage, neovascular glaucoma, and retinopathy of prematurity [30]. From a safety perspective, the most clinically relevant distinction is not simply between “safe” and “unsafe” agents, but between class-related injection complications, which are broadly similar across agents, and drug-specific safety signals, which appear more clearly with some molecules than others, particularly brolucizumab [29–33].

### A. Ocular safety

#### i. Endophthalmitis

One of the most serious adverse events after intravitreal injections is endophthalmitis. Although its incidence is decreasing, it is similar among different anti-VEGF medications, different geographical locations, and different injection settings [30]. Overall, current evidence suggests that endophthalmitis is primarily associated with injection technique and aseptic precautions rather than with the specific anti-VEGF agent administered [30].

Proper attention before, during, and after the intravitreal injection is the most crucial factor in reducing the risk of endophthalmitis. It is widely recommended to apply 5% povidone-iodine to the conjunctival folds as a preventive measure against endophthalmitis. Prophylactic topical antibiotics dramatically reduce ocular surface bacteria but do not significantly lower the risk of endophthalmitis. Following intravitreal anti-VEGF injection, surgeons should suspect that every case of uveitis is endophthalmitis, even if the presentation is more severe than in acute intraocular inflammation. When there are strong clinical suspicions, intravitreal antibiotics should be administered [34].

A randomized controlled study by Abouelkheir *et al*. involving 60 patients receiving bevacizumab, ranibizumab, or aflibercept found no significant differences in infectious endophthalmitis (*P* = 0.332 between groups) or ocular inflammation (*P* = 0.863 between groups) [35]. In addition, a systematic review and meta-analysis by Ma *et al*. using pharmacovigilance data showed the most prevalent adverse events associated with each agent, which were aflibercept with endophthalmitis (ROR = 178.27, IC-2SD = 6.70), raised intraocular pressure (ROR = 31.09, IC-2SD = 4.61), ranibizumab with retinal pigment epithelial tear (ROR = 836.54, IC-2SD = 7.19) and macular ischemia (ROR = 205.27, IC-2SD = 3.70), brolucizumab with retinal vascular occlusion (ROR = 391.11, IC-2SD = 6.10) and/or retinal vasculitis (ROR = 2930.41, IC-2SD = 7.47) and dry eye (ROR = 12.48, IC-2SD = 2.88) [31]. Moreover, a systematic review and meta-analysis by Samaća *et al*. on the safety of faricimab showed 74% fewer serious ocular adverse events than brolucizumab (RR = 0.26; 95% CI, 0.08–0.85), with no excess of serious ocular adverse events compared with other comparators [36]. In addition, studies by Tadayoni *et al*. and Yen *et al*. in 2024 showed similar safety profiles for faricimab (16.3%), aflibercept (20%), and other agents (OR = 1.10; 95% CI, 0.81–1.49) [37,38]. On the other hand, studies by Baumal *et al*. and Monés *et al*. on brolucizumab showed an increased risk of intraocular inflammation (19% and 4.6%, respectively) compared with other agents [29,32], as well as an increased risk of retinal artery occlusion (2.1%) and retinal vasculitis (3.3%) [29]. Moreover, compared with ranibizumab and aflibercept, brolucizumab was associated with more severe ocular adverse events ((OR = 2.15; 95% CI, 1.11–4.16; *P* = 0.02) [33]. Taken together, these findings suggest that whereas endophthalmitis remains largely a procedure-related complication across anti-VEGF agents, inflammatory ocular events and occlusive retinal vasculopathy represent a more distinctive safety concern with brolucizumab [29,32,33].

#### ii. Retinal detachment

Compared with endophthalmitis, the incidence of rhegmatogenous retinal detachment (RRD) and retinal tears after anti-VEGF intravitreal injection is relatively low (0-0.67%). RRD after anti-VEGF injections has been attributed either to the induction of posterior vitreous detachment or to improper injection technique, and it can be prevented using a smaller needle gauge, the proper injection site (3.5−4 mm posterior to the limbus), and avoiding vitreous reflux or wick by tunneled needle insertion [30]. Thus, retinal detachment appears to be an uncommon complication, primarily related to the injection procedure rather than to any specific anti-VEGF molecule [30]. A systematic review, meta-analysis, and trial sequential analysis on the efficacy and safety of faricimab showed an increased risk of vitreous floaters (OR = 1.76; 95% CI, 1.10–2.81; I2 = 0%) [39]. With regard to brolucizumab, the most commonly reported ocular adverse events included vitreous detachment (2.9%), punctate keratitis (2.6%), cataract (2.6%), and conjunctival hemorrhage (2.6%) [40]. Overall, available evidence does not indicate major inter-agent differences in retinal detachment risk, although the accompanying pattern of ocular adverse events may vary across agents.

#### iii. Ocular hemorrhage

Following anti-VEGF injections, subconjunctival hemorrhage has been reported, especially in patients receiving aspirin and, in particular, choroidal hemorrhage/detachment after bevacizumab, and subretinal hemorrhage following bevacizumab and ranibizumab [30]. Systematic reviews and meta-analyses on the safety and efficacy of intravitreal bevacizumab and aflibercept reported conjunctival hemorrhage as the most frequently reported adverse event (6.5%), mostly related to the injection procedure [41]. This suggests that ocular hemorrhagic events are generally minor, procedure-related, and of limited value in distinguishing the relative safety profiles of the different anti-VEGF agents.

#### iv. Intraocular pressure elevation

An acute rise in intraocular pressure (IOP) following intravitreal injections is expected and typically lasts a few hours. Moreover, patients with pre-existing glaucoma experience higher rates of IOP elevation than those without the condition [30]. In a study conducted by Abouelkheir *et al*., there were no significant differences between the three medications, bevacizumab, ranibizumab, and aflibercept, with regard to IOP elevation (*P* = 0.708) [35]. Therefore, transient IOP elevation appears to be a class effect of intravitreal delivery rather than a major drug-specific safety difference, although closer monitoring may be warranted in patients with glaucoma or ocular hypertension [30,35].

### B. Systemic safety

Systemic anti-VEGF monoclonal antibody administration has been associated with several adverse events, including thromboembolic events, stroke, myocardial infarction, hypertension, kidney disease, and gastrointestinal perforations [30]. This has raised concern that intravitreal anti-VEGF therapy, particularly with agents showing greater systemic persistence, might also be associated with clinically meaningful systemic toxicity. However, ophthalmic literature has generally not demonstrated large or consistent differences between agents. In one study, there were no significant differences among bevacizumab, ranibizumab, and aflibercept in systemic adverse events, whether cardiovascular or cerebrovascular (*P* = 0.56), or in changes in glomerular filtration rate (GFR) pre-injection (*P* = 0.784) or post-injection (*P* = 0.768) [35]. A systematic review and network meta-analysis of direct comprehensive studies concluded that ocular adverse events, some serious systemic adverse events, and the incidence rate ratio of death did not differ significantly between bevacizumab, ranibizumab, and aflibercept, although the bevacizumab group only had significantly higher serious systemic adverse events (*P* = 0.035) [42]. Another systematic review and meta-analysis evaluating bevacizumab and ranibizumab found no significant differences in adverse events, including incidence of death from all causes, hypertension, venous thrombotic events, vascular death, nonfatal myocardial infarction, stroke, and arteriothrombotic events, with pooled RRs being 1.11 (0.77, 1.61), 1.02 (0.29, 3.62), 2.38 (0.94, 6.04), 1.24 (0.63, 2.44), 0.97 (0.48, 1.96), 0.84 (0.39,1.80), 1.03 (0.69,1.55), respectively [43,44], except for gastrointestinal disorders which were higher in the bevacizumab group (RR = 1.82; 95% CI, 1.04–3.19; *P* = 0.04) [45]. A further systematic review and meta-analysis from 2024 examined the efficacy and safety of ranibizumab and aflibercept in the management of retinal vein occlusion and found no significant differences in their safety profiles [43]. A patient-level pooled analysis by Zarbin *et al*. concluded that there were no significant differences between ranibizumab and sham or verteporfin regarding arterial thromboembolic events [HR = 1.16 (0.72–1.88)], myocardial infarction [HR = 1.33 (0.59–2.97)], stroke [HR = 1.43 (0.54–3.77)], or vascular death [HR = 0.57 (0.18–1.78)] [44]. Moreover, a systematic review and meta-analysis on the safety of faricimab identified an increased risk of cough (OR = 2.00; 95 % CI, 1.02–3.91; I^2^=0 %) and protection against dyspnea (OR = 0.19; 95 % CI, 0.04–0.88; I^2^ =0 %) [39]. The limited number of events and the uncertainty of the clinical relevance of these signals demand caution in interpreting these findings. Considering the current evidence, systemic adverse events are still a legitimate, although largely theoretical, concern with anti-VEGF therapy; however, the robustness of agent-specific comparative differences has not been consistently proven. Table 2 summarizes the systemic adverse events associated with the anti-VEGF medications discussed.

**Table 2:** Systemic adverse events for anti-VEGF medications

Cardiovascular events	Cerebrovascular events	GFR* pre and post injection	Gastrointestinal disorders	Respiratory system
No significant differences between bevacizumab, ranibizumab, and aflibercept			Higher incidence with bevacizumab	Faricimab caused increased cough and protection against dyspnea
No significant differences between ranibizumab and sham or verteporfin				

This table outlines systemic adverse events associated with anti-VEGF medications, highlighting differences in cardiovascular, cerebrovascular, non-ocular hemorrhage, gastrointestinal, respiratory, and urinary tract infections among medications like bevacizumab, ranibizumab, aflibercept, and faricimab. **Abbreviations:** GFR, Glomerular Filtration Rate

### Cost implications of different anti-VEGF medications

This section analyzes anti-VEGF therapy and its economic impacts for wet age-related macular degeneration (wAMD), focusing on how cost-effectiveness outcomes vary across healthcare systems, pricing models, dosing schedules, and the assumptions underlying the analyses. The studies located are from various jurisdictions and utilized different currencies, time spans, and models, so the findings are likely context-specific rather than universally applicable.

Bevacizumab has consistently been identified in several studies as the most cost-effective treatment option for wAMD with an annual cost of approximately €27,087 and a change in visual acuity of 5.4 Early Treatment Diabetic Retinopathy Study (ETDRS) letters, compared to aflibercept with an annual cost of €31,119, about €4,000 more than bevacizumab, and a change in visual acuity of 8.4 letters. Ranibizumab costs around €33,137 per year, about €6,000 more than bevacizumab, with a change in visual acuity of 4.7 letters. Incremental cost-effectiveness ratios (ICERs) indicate that aflibercept costs €278,099 per quality-adjusted life year (QALY) gained compared to bevacizumab. Ranibizumab is dominated by bevacizumab, meaning it is more expensive and offers no additional health benefits. Therefore, for aflibercept to be cost-effective, the price should be reduced to €533 per injection, indicating that it is currently overpriced by €410 per injection. Aflibercept would need to provide an additional 10.3 letters of visual gain compared to bevacizumab to justify its higher cost [22]. Other studies comparing bevacizumab, ranibizumab, and aflibercept found that bevacizumab should be the first-line treatment for wAMD due to its superior cost-effectiveness. In an 11-year analysis, bevacizumab and ranibizumab each provided a gain of 1.339 quality-adjusted life years (QALYs), while aflibercept provided a gain of 1.380 QALYs. Aflibercept was more effective than ranibizumab but not as cost-effective as bevacizumab. Cost-utility ratios in this study were $11,033, $79,600, and $44,801 per QALY for bevacizumab, ranibizumab, and aflibercept, respectively [46]. From a societal perspective, the lowest annual treatment cost was for bevacizumab, whereas aflibercept and ranibizumab had higher costs and less favorable incremental cost-effectiveness ratios [22]. A cost-utility analysis over 11 years also found that bevacizumab and ranibizumab were similar in QALY gains, whereas aflibercept had a slightly higher QALY gain than ranibizumab but was still less economically favorable than bevacizumab [46]. These findings imply that, although bevacizumab has often been the economically preferred option in wAMD, this conclusion varies with local pricing, compounding legislation, and reimbursement policy.

Compared with bevacizumab, faricimab resulted in a mean gain of 0.05 QALYs with 46% fewer injections, implying a lower treatment burden for patients and lower healthcare costs. However, it incurred higher costs compared to bevacizumab by 11,987 Canadian Dollars (CAD). The incremental cost-effectiveness ratio (ICER) for faricimab compared with bevacizumab was CAD 226,373 per QALY gained, meaning it costs this amount to gain one additional QALY with faricimab compared with bevacizumab. The ICER per injection provided was CAD 222, indicating the cost-effectiveness of reducing the number of injections [47]. Faricimab appeared more cost-effective in that jurisdiction than ranibizumab, aflibercept, and brolucizumab, especially under typical treatment regimens. This is evident from the mean QALY gains for faricimab compared with ranibizumab (0.03 QALYs), aflibercept (0.05 QALYs), and brolucizumab (0.06 QALYs). Faricimab also required 37% fewer injections than ranibizumab, 21% fewer than aflibercept, and 28% fewer than brolucizumab. Overall, the analysis showed that faricimab led to lower costs than ranibizumab, aflibercept, and brolucizumab, resulting in savings of CAD 76,496, 29,117, and 38,235, respectively. Its ability to significantly reduce injection frequency enhances its cost-saving potential, making it a favorable option in clinical practice [48]. Recent studies have analyzed whether new agents justify higher procurement costs by considering diminishing injection burden. In a Canadian cost-effectiveness analysis of faricimab (an injectable treatment given every 4 weeks), it produced a slight improvement in QALYs and fewer injections than bevacizumab (another injectable treatment given every 4 weeks), but at much higher costs [47]. Within the same framework, faricimab was found to be more cost-effective than ranibizumab, aflibercept, and brolucizumab, mainly because a lower injection frequency reduced the long-term treatment burden and total costs. However, the model is highly sensitive to assumptions about the interval between doses, clinic visits, and use, drug prices, and the organization of the healthcare system, and thus the results should be applied cautiously in other regions.

Brolucizumab costs $63,614 and provides 4.580 QALYs. Aflibercept costs $72,189 and provides 4.572 QALYs. Ranibizumab costs $128,163 with 4.572 QALYs. Hence, brolucizumab is less costly and more effective than both aflibercept and ranibizumab [49]. A 2021 study also described brolucizumab as a cost-effective option compared with aflibercept and ranibizumab, based on an analysis showing lower total cost and a marginally greater QALY [49]. However, one must consider potential ocular safety issues with the drug, such as intraocular inflammation, retinal vasculitis, and retinal vascular occlusion, which could affect real-world treatment choices and downstream healthcare utilization [29,32,33].

Regarding pegaptanib, its lower efficacy results in a higher cost per QALY gained than for ranibizumab and aflibercept. Pegaptanib was withdrawn from the market in 2018 due to declining use and the availability of more effective treatments. Studies conducted prior to its withdrawal found pegaptanib to be not cost-effective compared with alternatives, primarily because the benefits in vision preservation did not justify its costs [50]. Pegaptanib now has limited relevance in contemporary economic comparisons because its narrower mechanism of action and lower clinical efficacy have diminished its role compared with later anti-VEGF agents. Any economic conclusions regarding pegaptanib should therefore be interpreted primarily in historical context rather than as part of the modern therapeutic landscape.

The economic comparisons discussed here are presented in Table 3. Based on the literature, bevacizumab appears to be the most consistently cost-effective option across many situations, whereas cost-effectiveness comparisons for aflibercept, ranibizumab, faricimab, and brolucizumab are more sensitive to local pricing, dosing assumptions, reimbursement structures, and modeled treatment burden. Pegaptanib has mainly historical relevance in this context.

**Table 3:** Efficacy and cost-effectiveness of anti-VEGF treatments for wAMD

Drug	Annual cost	Visual acuity improvement	ICER	Conclusion
**Ranibizumab**	€33,137	4.7 letters	€4,251.60 per QALY	Effective but costly
**Bevacizumab**	€27,087	5.4 letters	N/A	Most cost-effective first-line treatment
**Aflibercept**	€31,119	8.4 letters	€278,099 per QALY	High cost without significant benefits
**Brolucizumab**	$63,614 (lifetime)	6.9 letters	N/A	Cost-saving and effective
**Faricimab**	N/A	7 letters	£8,898 per QALY	Dominant in effectiveness and cost savings
**Pegaptanib**	$8,839	Lower than others	N/A	Not cost-effective, withdrawn from the market

This table compares annual costs, visual acuity improvements, and ICER for various anti-VEGF treatments, highlighting their effectiveness and cost considerations in managing wet age-related macular degeneration. **Abbreviations:** ICER, Incremental Cost-Effectiveness Ratio, a measure used to assess the cost-effectiveness of a health care intervention; QALY, Quality-Adjusted Life Year, a measure used to assess the value of health outcomes; €, Euro; $, Canadian dollar; £, Great British Pound

Interpretation of these cost-effective findings should be undertaken with caution, as the included studies were conducted across different healthcare systems and jurisdictions. Variations in drug pricing, reimbursement policies, treatment protocols, healthcare resource utilization, and perspectives adopted in economic evaluations limit the direct comparability of cost and ICER estimates across settings. Additionally, differences in currency, time horizons, and assumptions regarding injection frequency and monitoring may influence reported cost-effectiveness outcomes. As a result, these findings may not be directly generalizable to all healthcare systems and should be interpreted within the context of local economic and clinical practice conditions.

## CONCLUSION

In summary, faricimab represents a significant advancement in the treatment of diabetic macular edema and neovascular age-related macular degeneration by dual targeting of the VEGF-A and Ang-2 pathways. Its unique design, long-lasting efficacy, and reduced dosing frequency make it a promising alternative to conventional anti-VEGF therapies. Alongside other medications such as brolucizumab, pegaptanib, aflibercept, ranibizumab, and bevacizumab, faricimab contributes to a diverse arsenal of treatments for retinal disorders, each with distinct mechanisms, structures, and dosing regimens. This diversity supports a more individualized approach to treatment selection based on clinical indication, treatment burden, and patient-specific factors. However, despite the effectiveness of intravitreal anti-VEGF treatments, they are associated with ocular and systemic adverse events, ranging from endophthalmitis and retinal detachment to IOP elevation and systemic thromboembolic risks. Current evidence suggests that many adverse events are related to the injection procedure itself, whereas some molecule-specific safety signals, particularly with brolucizumab, warrant closer attention. These findings highlight the importance of careful patient selection, meticulous injection technique, adherence to antiseptic protocols, and appropriate post-injection monitoring. In terms of cost, bevacizumab stands out as the most cost-effective treatment for wAMD, offering significant savings without compromising clinical outcomes. While aflibercept and ranibizumab demonstrate some efficacy, their higher costs lead to considerable overspending, making them less favorable as first-line options. Faricimab and brolucizumab present promising alternatives, with improved cost-effectiveness driven by reduced injection frequencies and lower overall treatment costs, particularly compared with aflibercept and ranibizumab. Pegaptanib, due to its lower efficacy and cost-utility, has been withdrawn from the market, underlining the importance of ongoing evaluation and optimization of treatment strategies for wAMD. These findings underscore the need for a patient-centered approach that balances cost, clinical effectiveness, and treatment burden in decision-making.
